# Glioblastoma—A Contemporary Overview of Epidemiology, Classification, Pathogenesis, Diagnosis, and Treatment: A Review Article

**DOI:** 10.3390/ijms262412162

**Published:** 2025-12-18

**Authors:** Kinga Królikowska, Katarzyna Błaszczak, Sławomir Ławicki, Monika Zajkowska, Monika Gudowska-Sawczuk

**Affiliations:** 1 Department of Population Medicine and Lifestyle Diseases Prevention, The Faculty of Medicine, Medical University of Bialystok, 15-269 Bialystok, Poland; k.krolikowska@gmail.com (K.K.); katarzyna.blaszczak@sd.edu.pl (K.B.); slawicki@umb.edu.pl (S.Ł.); 2 Department of Neurodegeneration Diagnostics, Medical University of Bialystok, Waszyngtona 15A St., 15-269 Bialystok, Poland; monika.zajkowska@umb.edu.pl; 3 Department of Biochemical Diagnostics, Medical University of Bialystok, Waszyngtona 15A St., 15-269 Bialystok, Poland

**Keywords:** glioblastoma multiforme, IDH-wildtype, molecular diagnostics, radiogenomics, immunotherapy, WHO CNS5 classification

## Abstract

Glioblastoma (GBM) is one of the most common and aggressive primary malignant tumors of the central nervous system, accounting for about half of all gliomas in adults. Despite intensive research and advances in molecular biology, genomics, and modern neuroimaging techniques, the prognosis for patients with GBM remains extremely poor. Despite the implementation of multimodal treatment involving surgery, radiotherapy, and chemotherapy with temozolomide, the average survival time of patients is only about 15 months. This is primarily due to the complex biology of this cancer, which involves numerous genetic and epigenetic abnormalities, as well as a highly heterogeneous tumor structure and the presence of glioblastoma stem cells with self renewal capacity. Mutations and abnormalities in genes such as IDH-wt, EGFR, PTEN, TP53, TERT, and CDKN2A/B are crucial in the pathogenesis of GBM. In particular, IDH-wt status (wild-type isocitrate dehydrogenase) is one of the most important identification markers distinguishing GBM from other, more favorable gliomas with IDH mutations. Frequent EGFR amplifications and TERT gene promoter mutations lead to the deregulation of tumor cell proliferation and increased aggressiveness. In turn, the loss of function of suppressor genes such as PTEN or CDKN2A/B promotes uncontrolled cell growth and tumor progression. The immunosuppressive tumor microenvironment also plays an important role, promoting immune escape and weakening the effectiveness of systemic therapies, including immunotherapy. The aim of this review is to summarize the current state of knowledge on the epidemiology, classification, pathogenesis, diagnosis, and treatment of glioblastoma multiforme, as well as to discuss the impact of recent advances in molecular and imaging diagnostics on clinical decision-making. A comprehensive review of recent literature (2018–2025) was conducted, focusing on WHO CNS5 classification updates, novel biomarkers (IDH, TERT, MGMT, EGFR), and modern diagnostic techniques such as liquid biopsy, radiogenomics, and next-generation sequencing (NGS). The results of the review indicate that the introduction of integrated histo-molecular diagnostics in the WHO 2021 classification has significantly increased diagnostic precision, enabling better prognostic and therapeutic stratification of patients. Modern imaging techniques, such as advanced magnetic resonance imaging (MRI), positron emission tomography (PET), and radiomics and radiogenomics tools, allow for more precise assessment of tumor characteristics, prediction of response to therapy, and monitoring of disease progression. Contemporary molecular techniques, including DNA methylation profiling and NGS, enable in-depth genomic and epigenetic analysis, which translates into a more personalized approach to treatment. Despite the use of multimodal therapy, which is based on maximum safe tumor resection followed by radiotherapy and temozolomide chemotherapy, recurrence is almost inevitable. GBM shows a high degree of resistance to treatment, which results from the presence of stem cell subpopulations, dynamic clonal evolution, and the ability to adapt to unfavorable microenvironmental conditions. Promising preclinical and early clinical results show new therapeutic strategies, including immunotherapy (cancer vaccines, checkpoint inhibitors, CAR-T therapies), oncolytic virotherapy, and Tumor Treating Fields (TTF) technology. Although these methods show potential for prolonging survival, their clinical efficacy still needs to be confirmed in large studies. The role of artificial intelligence in the analysis of imaging and molecular data is also increasingly being emphasized, which may contribute to the development of more accurate predictive models and therapeutic decisions. Despite these advancements, GBM remains a major therapeutic challenge due to its high heterogeneity and treatment resistance. The integration of molecular diagnostics, artificial intelligence, and personalized therapeutic strategies that may enhance survival and quality of life for GBM patients.

## 1. Introduction

Gliomas are the most common primary tumors of the central nervous system, among which glioblastoma (GBM) is the most aggressive and deadly form, accounting for 60% of all brain tumors in adults [1]. The current classification of the World Health Organization (WHO, 2021) has significantly increased the role of molecular markers in defining tumor subtypes, leading to a redefinition of the term “glioblastoma”, which refers to high-grade tumors that meet IDH-wildtype criteria and specific molecular and histological characteristics [2]. This change has important implications for diagnosis, prognosis, and clinical trial design [3].

In epidemiological terms, glioblastoma is the most common primary malignant brain tumor in adults, accounting for approximately 45–50% of all gliomas [4]. The global incidence of this disease varies significantly between countries and continents. The highest incidence rates are found in highly developed countries—in the United States (US), an average of 3.19 cases per 100,000 individuals per year, and in Australia, approximately 3.4/100,000 [5]. In Europe, these figures are similar to those in the US, although there are slight regional differences between northern and southern countries [6]. Recent population-based analyses indicate that overall incidence trends have remained relatively stable over the past decade, with a slight upward tendency in aging populations [5]. Moreover, demographic disparities are evident: higher incidence is reported among individuals of White ethnicity compared with Black or Asian populations [7], and socioeconomic differences continue to influence both diagnosis rates and outcomes [8]. The high incidence in developed countries may be partly related to better admittance to diagnostic imaging, higher life expectancy, and more complete case reporting, while in lower-income regions, the actual incidence may be underestimated due to underdiagnosis and limited access to the neurooncological care [1].

In terms of demographics, GBM most commonly occurs in individuals aged 55–75, with the peak incidence in the sixth decade of life [9]. The disease also shows gender differences, men are affected about 50% more often than women, which may be related to hormonal alterations, differences in the expression of X-linked genes, and environmental factors [10]. In children, glioblastoma multiforme is rare and has a different genetic profile, suggesting a different pathogenesis mechanism [11]. It is also important to emphasize that, according to the WHO CNS5 classification, the term “glioblastoma” applies exclusively to adult-type diffuse gliomas that are IDH-wildtype, while pediatric high-grade gliomas constitute a separate group defined by distinct molecular alterations [12]. Consequently, tumors formerly diagnosed as pediatric GBM are now classified under pediatric-type diffuse high-grade gliomas, which differ in epidemiology, biology, and prognosis from adult GBM [2].

Clinically, GBM remains a cancer with an extremely poor prognosis. Despite advances in imaging and molecular diagnostics and the introduction of integrated treatment involving maximum surgical resection, radiotherapy, and chemotherapy with temozolomide, the median survival of patients is approximately 14–18 months, and the five year survival rate does not exceed 5% [9]. Recurrence of the disease is almost inevitable and poses a significant clinical problem, resulting, among other causes, from the presence of cancer stem cells, resistance to treatment, and limited penetration of drugs through the blood–brain barrier [13].

The etiology of GBM remains largely unclear. Potential risk factors include previous exposure to ionizing radiation, immunosuppression, genetic predisposition (including Li-Fraumeni syndrome, Turcot syndrome, type 1 neurofibromatosis), and environmental factors [14,15]. Based on the above findings, glioblastoma multiforme remains one of the greatest challenges in modern neurooncology. The aim of this review is to present the current state of knowledge on the etiopathogenesis, molecular classification, epidemiology, diagnosis, and therapeutic strategies (both available and developing) for GBM. In addition, the article identify key directions for future research that could help improve patient prognosis and quality of life in this exceptionally malignant cancer.

## 2. Pathogenesis and Molecular Biology

In the last few years, the combination of molecular data and morphology has resulted in significant improvements in the classification of central nervous system (CNS) malignancies. Epigenetics plays a key role in shaping the complex biology of gliomas, and its impact extends far beyond the traditionally discussed MGMT promoter methylation. Recent studies have shown that global patterns of DNA methylation, histone modifications, and disruptions of chromatin architecture constitute a distinct layer of tumor regulation that significantly influences intratumoral heterogeneity, glioma cell plasticity, and clonal evolution [16]. The chemical classification of DNA methylation has made it possible to identify unique molecular subtypes of glioblastoma—including classical, proneural, mesenchymal, and neural—each with its own epigenetic landscape and gene expression profile. This approach has highlighted that epigenetics not only passively reflects the tumor state but actively shapes it by driving, among other processes, the phenotypic switching of tumor cells between proneural and mesenchymal states—one of the mechanisms underlying clinical aggressiveness and therapeutic resistance [17].

Alterations in DNA methylation and histone modifications modulate the activity of key pathways such as EGFR, PI3K/AKT, Notch, and Wnt, thereby regulating the behavior of tumor cells and glioblastoma stem cells (GSCs). Moreover, epigenetic dysregulation affects interactions between tumor cells and the microenvironment, for example by controlling the transcription of cytokines, chemokines, and immune checkpoint ligands, contributing to the immunosuppressive nature of the GBM TME [18,19]. Methylation patterns are stable, heritable, and at the same time responsive to environmental cues, making them a central mechanism driving dynamic phenotypic plasticity and, consequently, the development of treatment resistance.

In the context of glioblastoma heterogeneity, single-cell sequencing data have been particularly transformative, reshaping our understanding of the tumor’s internal architecture over the last decade. Single-cell RNA-seq studies demonstrated that GBM consists of multiple coexisting subpopulations, differing in transcriptional profiles, metabolic states, degrees of differentiation, and vulnerabilities to therapy [20,21]. Importantly, these populations respond to therapeutic pressure in markedly different ways, and treatment frequently leads to selective expansion of resistant clones. Further studies have confirmed the presence of a continuous spectrum of cellular states, rather than sharply defined subtypes, suggesting that GBM heterogeneity is dynamic and fluid, regulated by an intertwined epigenetic–transcriptional network [22,23]. Chen et al. additionally observed that macrophage subpopulations within the tumor may undergo epigenetically driven EZH2 activation, promoting pro-tumor phenotypes and further destabilizing the tumor’s cellular landscape [24].

When integrated with methylation analyses and multi-regional tumor sampling, single-cell data describe GBM clonal evolution as a nonlinear process involving the simultaneous branching of subclones, their cooperation, and occasionally, phenotypic convergence [25,26]. This gives rise to distinct ecological niches within a single tumor mass, including hypoxic zones, perivascular regions, and areas enriched in GSCs, all of which critically influence tumor progression and therapeutic resistance [27,28,29].

**Figure 1:**
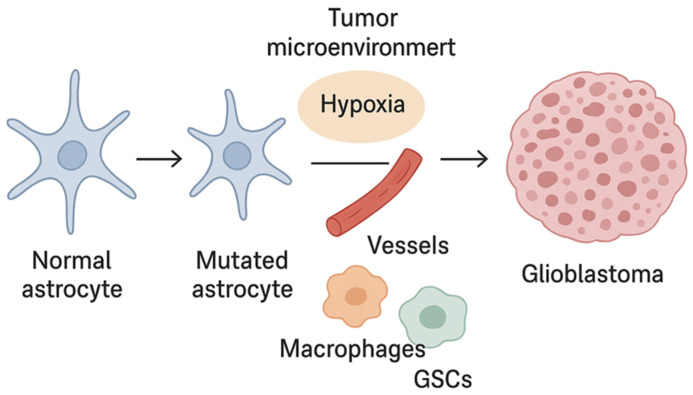
Glioblastoma development.

The contemporary model of glioblastoma pathogenesis therefore assumes a tight correlation between genetic mutations (EGFR, PTEN, TP53, TERT), epigenetics, and microenvironmental niches, which synergistically define tumor biology, aggressiveness, recurrence potential, and treatment resistance. Integrating epigenetics into practical tumor classification (as proposed in recent work on DNA-methylation-based CNS tumor classes) aligns with the WHO CNS5 framework, which emphasizes the need for multidimensional data integration to improve diagnostic and prognostic precision [30,31] (Figure 1).

## 3. Clinical Picture

The clinical picture of glioblastoma multiforme (GBM) is varied and depends on the location of the tumor, its growth rate, and its massive effects. Patients most often present with: focal symptoms such as limb weakness, speech impairment (aphasia), visual disturbances, and seizures occur depending on the part of the brain affected by the tumor; damage to motor areas leads to muscle weakness, while involvement of speech centers leads to communication difficulties; symptoms of increased intracranial pressure, as headaches, nausea, vomiting, and impaired consciousness are the result of increased pressure within the skull caused by tumor growth and brain swelling; or cognitive and behavioral changes in which many patients experience memory impairment, personality changes, disorientation, and psychomotor retardation, reflecting the tumor’s impact on higher brain functions [32,33]. The course of the disease is characterized by a high tendency for local infiltration, rapid growth, and resistance to treatment, which means that despite aggressive therapy, relapses are almost inevitable [34]. Table 1 summarizes the most common clinical manifestations and the typical course of glioblastoma (GBM).

Glioblastoma multiforme manifests a wide spectrum of neurological disorders, the nature of which depends on the location and extent of the tumor. The disease progresses very aggressively, and despite advances in diagnosis and treatment, the prognosis remains poor. Rapid diagnosis and an interdisciplinary approach are crucial to improving patients’ quality of life [32,35,37].

## 4. Diagnosis

The diagnosis of glioblastoma multiforme (GBM) is one of the most important stages in clinical management, determining both the therapeutic strategy and the patient’s prognosis. Due to its high biological heterogeneity and aggressive course, the diagnosis of GBM requires an integrated approach combining classical imaging methods, histopathological analyses, and advanced molecular techniques [2].

In recent years, neurooncological diagnostics have undergone a significant transformation, from traditional morphological criteria to a complex molecular-genetic profile. According to the 2021 WHO classification, changes have been introduced, emphasizing the need to include genetic markers such as TERT mutations, IDH status, and MGMT promoter methylation [38]. Today, the diagnosis of GBM is therefore based on a so-called integrated diagnostic model, combining histological, immunohistochemical, molecular, and imaging data [13]. At the same time, the development of non-invasive technologies such as liquid biopsy, radiogenomics, and next-generation sequencing (NGS) allows for assessment of minimal residual disease (MRD), increasingly accurate monitoring of disease progression, and importantly earlier detection of recurrence [32].

### 4.1. Imaging Diagnostics (MRI, fMRI, PET)

Currently, magnetic resonance imaging (MRI) remains the gold standard in GBM diagnosis, enabling the assessment of tumor location, margins and the nature of intracerebral lesions [39]. In a typical MRI image, the tumor is characterized by irregular contrast enhancement, central necrosis, and peritumoral edema. In turn, advanced techniques such as perfusion MRI (DSC-MRI, DCE-MRI), diffusion MRI (DWI, DTI), and proton spectroscopy (^1^H-MRS) provide additional information on angiogenesis, cell proliferation, and tumor metabolism. Furthermore, functional MRI (fMRI) and tractography (DTI) are valuable adjunctive tools in neurosurgical planning, allowing maximal tumor resection while preserving neurological function [40,41]. Positron emission tomography (PET), particularly with amino acid radiotracers (^11^C-methionine, ^18^F-FDOPA), is used to differentiate tumor recurrence from radiation-induced changes and necrosis [42]. It is also worth emphasizing that currently, hybrid PET/MRI systems and artificial intelligence-assisted radiomics analyses are increasingly used to assess tumor heterogeneity and early prediction of treatment response [43].

### 4.2. Molecular and Histopathological Diagnostics—The Importance of Biomarkers

Molecular and histopathological diagnosis of GBM has undergone a profound transformation in recent years, resulting from the new WHO classification of 2021, which introduced an integrated histopathological-molecular approach to the diagnosis of central nervous system tumors [2]. According to current guidelines, the diagnosis of GBM requires not only morphological evaluation, but also confirmation of characteristic genetic changes that define the tumor phenotype and have important prognostic and predictive significance [44].

According to the WHO CNS5 classification, glioblastoma (IDH-wildtype) is diagnosed in cases of high-grade astrocytic tumors. These tumors do not exhibit mutations in the IDH1 or IDH2 genes and at the same time have at least one of three key molecular aberrations: TERT promoter mutation, EGFR amplification or loss of heterozygosity in chromosomes 10q and 7p (the so-called +7/−10 signature) [45,46]. Tumors with IDH mutation are classified as astrocytoma, IDH-mutant, grade 4, even if they show histopathological features of GBM. Importantly, the IDH1 mutation (most commonly R132H) is associated with younger age at onset, slower clinical course, and significantly better prognosis compared to IDH-wildtype tumors [47]. This tumor can be detected using immunohistochemistry (IHC) or molecular techniques such as PCR and Sanger sequencing [48,49]. Another biomarker of great clinical significance is methylation of the MGMT (O6-methylguanine-DNA methyltransferase) gene promoter, which leads to epigenetic silencing of this gene and reduces the ability of cancer cells to repair DNA damage caused by alkylating cytostatics such as temozolomide [50]. According to the EANO I NCCN guidelines, assessment of MGMT methylation status is currently recommended as a standard step in the diagnosis of GBM [51]. At the same time, recent studies highlight that molecular subtyping of GBM extends beyond the canonical genomic markers recommended by the WHO and increasingly incorporates tumor–immune microenvironment characteristics. Several modern classification frameworks demonstrate how integrating immune signatures with transcriptomic and epigenetic data can refine GBM stratification and improve prognostic and therapeutic precision. For example, Zhang et al. proposed an immune-based classification dividing GBM into distinct subgroups characterized by differential immune infiltration patterns, cytokine signaling activity, and expression of immunoregulatory genes, with each subtype showing unique survival trajectories and potential therapeutic vulnerabilities [52]. Similarly, Zheng et al. demonstrated that integrating immunogenomic profiles with classical molecular determinants (e.g., EGFR amplification, PTEN loss, TERT mutations) enables the identification of clinically meaningful GBM clusters that differ in tumor microenvironment composition, immune evasion pathways, and predicted response to immunotherapy [53]. These classification models underscore the increasing role of immune biology in molecular stratification and point toward a future in which GBM subtyping will rely on multidimensional profiling that combines genomic, epigenetic, and immunological determinants to guide individualized therapeutic strategies.

Histopathological diagnosis still plays a key role in the diagnosis of GBM. The microscopic image is characterized by the presence of cells with pronounced pleomorphism and nuclear atypia, a high number of mitoses, increased microvascular proliferation and palisading necrosis [54]. However, in light of current recommendations, histology is only a starting point—the final classification is based on the integration of histological and molecular data (so-called integrated diagnosis). In diagnostic practice, immunohistochemical panels are currently used, including markers such as GFAP, OLIG2, ATRX, p53, Ki-67, and IDH1-R132H, which enable the differentiation of gliomas and a preliminary assessment of their malignancy [55,56].

### 4.3. Liquid Biopsy

One of the most innovative, minimally invasive diagnostic tools that is gaining increasing importance in neurooncology, including the diagnosis and monitoring of glioblastoma multiforme, is liquid biopsy. In contrast to classical tissue biopsy, this method is based on the analysis of tumor biomarkers present in body fluids, primarily in plasma and cerebrospinal fluid (CSF) [57]. The basic premise of liquid biopsy is to detect and analyze circulating tumor-derived molecules, such as circulating tumor DNA (ctDNA), RNA (cfRNA), circulating tumor cells (CTCs), microRNA (miRNA), exosomes, and other extracellular microparticles [57]. These elements may reflect the current molecular state of the tumor and its microenvironment, enabling both early detection and monitoring of disease progression and treatment resistance [58]. Of particular importance in the case of GBM is the analysis of ctDNA and tumor-derived exosomes, which contain DNA, RNA, and proteins characteristic of glioma cells. It has been revealed that mutations of the IDH1, EGFR, TERT, and PTEN genes can be detected in ctDNA, which can be used for both diagnosis and monitoring of treatment effects [59]. However, it is worth emphasizing that due to the presence of the blood–brain barrier, which limits the release of genetic material into the bloodstream, the amount of ctDNA in the plasma of patients with GBM is usually much lower than in other solid tumors, reducing assay sensitivity and highlighting the need for further methodological refinement to improve clinical applicability. For this reason, there is growing interest in the analysis of cerebrospinal fluid (CSF), which is in direct contact with tumor tissue and may be a more reliable source of molecular information [60].

### 4.4. Diagnostic Innovations

Technological advances in recent years have significantly expanded the possibilities for diagnosing glioblastoma multiforme, shifting the focus from classic histopathological methods to an integrated, multidimensional diagnostic approach that includes molecular, imaging, and bioinformatic analyses [61]. Modern diagnostic methods are moving towards the personalization of neurooncological medicine, enabling individual assessment of the genetic and biological profile of a tumor, prediction of response to treatment, and real-time monitoring of molecular changes.

Currently, radiogenomics is one of the most dynamically developing fields combining radiological imaging with genomic and epigenetic data. Models based on radiomic analysis, using machine learning, can identify imaging features that correlate with the molecular status of a tumor, such as IDH1 mutation, MGMT promoter methylation, or EGFR amplification [62]. Available literature data confirm that complex radiogenomic algorithms can predict the molecular subtype of glioma with very high accuracy (85–90%) based on standard MRI. This fact can be of great importance in prognosis and treatment planning, especially when biopsy is impossible or risky [63].

Artificial intelligence (AI)-assisted imaging technology has profoundly revolutionized neurooncology diagnostics, enabling highly precise yet automatic tumor segmentation, detection of subtle or subclinical structural and functional changes, and dynamic monitoring of disease progression [64]. Modern artificial intelligence systems, particularly those based on deep learning and convolutional neural networks are capable of analyzing multiparametric MRI data, including perfusion, diffusion and spectroscopy sequences, with very high accuracy that in many cases surpasses traditional radiological assessment [65]. This facilitates earlier identification of infiltrating tumor margins and treatment-related changes, allowing clinicians to distinguish true tumor progression from pseudoprogression or radiation necrosis, both of which traditionally presented significant diagnostic challenges [66]. Moreover, AI-driven radiomics and radiogenomics enable the extraction of thousands of quantitative imaging features—such as texture, heterogeneity, and morphological parameters—that are invisible to the human eye [67]. Integrating these features with patient-specific molecular and genetic profiles (e.g., IDH mutation status, MGMT promoter methylation, EGFR amplification, TERT promoter mutations) allows for non-invasive prediction of tumor biology, prognosis, and potential treatment response [68]. To fully capture the current scope of artificial intelligence research in GBM, it is important to include specific methodological examples of how multimodal datasets are integrated into unified analytical models. Modern AI-driven workflows typically involve several sequential steps: (1) preprocessing and harmonization of imaging data (MRI, perfusion, diffusion), (2) automated tumor segmentation using convolutional neural networks, (3) extraction of radiomic features describing tumor heterogeneity, morphology, texture, and perfusion patterns, (4) integration of these features with genomic, transcriptomic, methylation, or histopathology-derived data, and finally (5) construction of prognostic or predictive models using machine-learning or deep-learning algorithms. One representative example is the study by Zhang et al. who proposed an AI-based computational pipeline integrating MRI radiomics with molecular biomarkers to identify biologically meaningful signatures associated with treatment response and overall survival in GBM patients. Their approach demonstrated how radiogenomic features can be fused to support individualized therapeutic decision-making [69]. Similarly, Park et al. developed a deep-learning radiogenomic framework predicting key molecular alterations—including IDH mutation, MGMT methylation, and EGFR amplification—exclusively from imaging data, achieving high diagnostic accuracy and demonstrating the feasibility of non-invasive molecular profiling [70]. These studies illustrate how AI can support biomarker discovery, early risk stratification, personalized treatment planning, and the identification of therapeutic targets. Furthermore, longitudinal image analysis supported by artificial intelligence enhances clinicians’ ability to monitor tumor changes over time, enabling the creation of predictive models describing the dynamics of tumor progression and early adaptation of therapeutic strategies. In summary, incorporating AI-based imaging tools into routine clinical practice has significant potential to improve diagnostic accuracy, personalize treatment, and ultimately improve outcomes in patients with glioblastoma multiforme [71].

It should be noted that AI- and radiogenomic algorithms may be influenced by biases related to training dataset diversity, size, and demographic representation, which can affect model generalizability and reliability. These biases may arise when training data are predominantly from specific populations, imaging centers, or acquisition protocols, potentially limiting the algorithm’s performance in broader, more heterogeneous patient cohorts. Additionally, preprocessing steps, feature extraction methods, and heterogeneity of tumor biology (e.g., genetic and cellular diversity) can introduce variability that reduces model robustness. Addressing these limitations through the use of large, multicenter, demographically diverse datasets, standardized imaging protocols, and independent external validation cohorts is critical for reliable clinical translation [71].

Intraoperative techniques are another innovative group of diagnostic methods that allow for the rapid assessment of resection margins and the identification of tumor tissue during neurosurgical procedures. Among them, Raman spectroscopy occupies a special place, a technique that uses light scattering to analyze the biochemical composition of tissues It allows for the identification of cancer cells with over 90% accuracy, clearly distinguishing them from healthy cells and providing the neurosurgeon with real-time information during the procedure [72]. Equally promising are intraoperative fluorescence methods using 5-aminolevulinate (5-ALA), which selectively accumulates in cancer cells and allows visualization of the tumor in the surgical field. Combining this technique with hyperspectral imaging and confocal microscopy increases the precision of resection and minimizes the risk of leaving cancerous tissue behind [73,74].

Recent years have seen significant progress in rapid intraoperative molecular tests, including modern, accelerated NGS panels. These enable the precise identification of key genetic aberrations, such as IDH1, TERT, EGFRvIII mutations, and PTEN loss of function, often in less than 24 h [75,76]. Next-generation sequencing (NGS) is a high-throughput technique that allows simultaneous analysis of thousands of DNA fragments, facilitating the detection of a wide range of genetic alterations—from point mutations to copy number variations—in a single analytical workflow [77]. This method utilizes a step-by-step procedure involving DNA fragmentation, preparation of sequence libraries, their amplification, and parallel sequencing, which is then subjected to advanced bioinformatics processing. This complex yet rapid analysis enables high-resolution molecular profiling of tumor tissue in a very short time [78]. In the coming years, these solutions may provide the basis for dynamically adapting surgical or therapeutic strategies during the procedure, making real-time molecular decision-making not only possible but also increasingly clinically practical [77].

Another new and extremely promising direction is the development of single-cell sequencing technologies, which allow for the assessment of the genetic and transcriptomic heterogeneity of individual tumor cells [79]. Studies using this technique have shown the presence of diverse cell subpopulations with different gene expression profiles, which explains the phenomenon of treatment resistance and differences in progression dynamics [80]. It therefore appears that in the future, single-cell analysis may enable the creation of personalized therapeutic strategies targeting dominant tumor clones.

**Figure 2:**
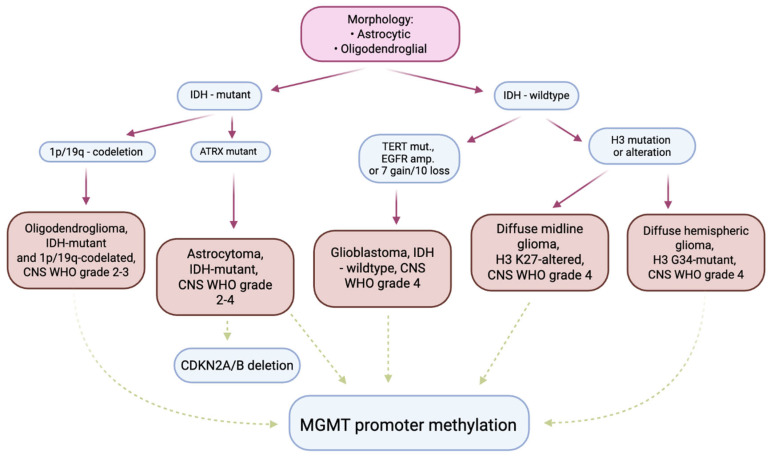
The diagnostic pathway used in the diagnosis of gliomas in adults—summary. The pink panel shows the microscopic methods used to classify the lesion. The blue panels show the molecular tests commonly used in diagnosis and prognosis. The burgundy arrows indicate diagnostic use. The green arrows indicate tests used in prognosis/staging/prediction of response to treatment. The orange panels indicate diagnostic entities as defined in the 2021 WHO Classification of Tumors of the Central Nervous System.

**Figure 3:**
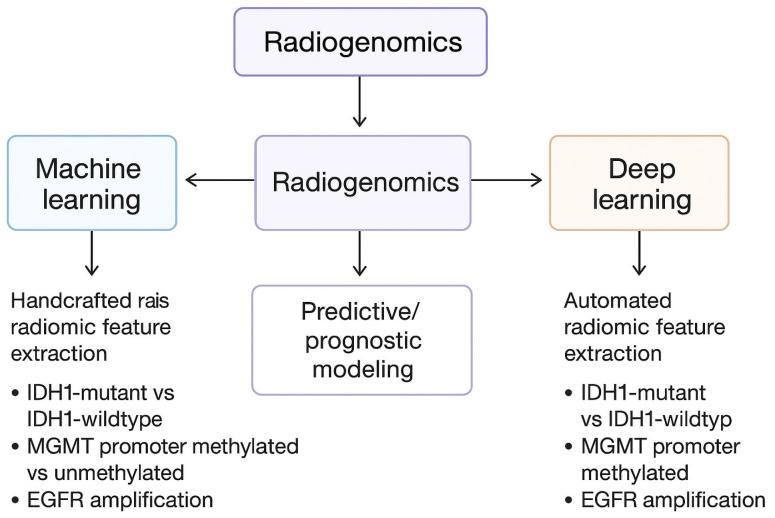
Division of AI/radiomics segments into machine learning and deep learning applications, correlating imaging features with molecular subtypes.

The diagrams below shows the exact diagnostic pathway along with the WHO classification of the tumor (Figure 2) and division of AI/radiomics segments into machine learning and deep learning applications, correlating imaging features with molecular subtypes (Figure 3).

## 5. Treatment

Glioblastoma multiforme remains a highly malignant tumor characterized by a short median survival. Multimodal treatment combining surgery, radiotherapy, and chemotherapy offers hope. In recent years, immunological and viral strategies, molecularly targeted therapies, and modern drug delivery technologies have been rapidly developing. All aimed at overcoming tumor heterogeneity and its highly immunosuppressive microenvironment. The first step in treatment remains a maximally safe resection, which reduces tumor mass and improves the volumetric effects of adjuvant therapies. However, the diffuse, infiltrative nature of GBM usually prevents cure with surgery alone. To date, the mainstay of first-line therapy after tumor resection is fractionated radiotherapy (usually 60 Gy/30 fractions) with concurrent and adjuvant temozolomide (Stupp protocol). In clinical practice, modifications (e.g., different doses, shorter treatment schedules in elderly patients) and combinations with new immunological methods are also being investigated. However, temozolomide remains the main systemic drug, usually providing greater benefit in patients with MGMT promoter methylation. Intensified chemotherapy has not yet replaced standard temozolomide in first-line treatment [68]. However, new therapies are known. Personalized neoantigen vaccines (peptide, mRNA, dendritic) have the ability to induce T-cell responses and local lymphocytic infiltration in GBM. Early clinical trials show promising signs of immunogenicity, although the impact on survival needs to be confirmed in randomized trials and depends on factors such as the use of glucocorticosteroids [81]. CAR-T cells targeting antigens such as IL13Rα2, EGFRvIII, or EGFR (in multitarget constructs) and administered intrathecally/directly into the CNS cavity show the ability to elicit rapid responses in some patients. The latest reports (2025) describe significant, though often transient, tumor regressions with acceptable toxicity, and intensive work is underway to add further targets and improve the durability of the effect [82,83]. BiTE (bispecific T cell activating) molecules, which bring T cells closer to tumor cells, may be a promising adjunct to targeted immunotherapy. Preliminary studies with EGFRvIII/EGFR demonstrate their activity in preclinical models and early-phase clinical trials [84]. Oncolytic adenoviruses (e.g., DNX-2401/Delta-24-RGD) and other vectors (PVSRIPO—oncolytic poliovirus-like virus) have passed phase I/II and, in combination with checkpoint inhibitors, show clinical signs of improved response in some patients; DNX-2401 + pembrolizumab showed increased response and improved 12-month survival in early phase studies. In short, oncolytics stimulate both direct lytic antitumor activity and secondary immune response [85]. Device-based therapies are also known—Tumour Treating Fields (TTFields). TTFields (low-intensity alternating electric field, clinical device “Optune”) added to the standard showed improved survival in initial studies, and real-world data and reviews from 2023 to 2025 confirm clinical utility in selected patients; issues related to treatment availability and compliance remain relevant [86]. Nanotechnologies and drug delivery systems, as well as stem cell-based therapies, offer new perspectives in the treatment of glioblastoma multiforme. Nanoparticles, liposomes, and other carrier systems are designed to cross the blood–brain barrier, selectively deliver cytostatics/oligonucleotides, and reduce systemic toxicity. Despite encouraging preclinical results, clinical outcomes remain limited by issues with the distribution, elimination, and immunogenicity of nanomaterials [85]. Neural and mesenchymal stem cells are being considered as carriers of prodrugs, gene vectors, or regenerative agents due to their tropism for tumor sites; safety aspects (transformation potential) and function control remain under investigation [87].

Recent studies have demonstrated that the heparanase inhibitor RDS 3337 significantly modulates the balance between apoptosis and autophagy in U87 glioma cells, providing novel insights into the molecular mechanisms governing cell death and potential therapeutic targets. Pharmacological inhibition of heparanase with RDS 3337 resulted in the accumulation of the lipidated form of LC3-II and elevated p62/SQSTM1 levels, indicative of impaired autophagic-lysosomal flux. Concomitantly, autophagy inhibition was associated with activation of apoptotic pathways, as evidenced by increased levels of cleaved caspase-3, the appearance of cleaved PARP1, and DNA fragmentation. These findings suggest that heparanase supports autophagic processes, and its inhibition can shift the cellular response toward apoptosis, potentially enhancing the susceptibility of cancer cells to anticancer therapies. Accordingly, heparanase inhibitors such as RDS 3337 represent a promising strategy to modulate cell death pathways and control tumor progression by regulating the interplay between autophagy and apoptosis [88].

Immunotherapy for glioblastoma (GBM) has generated considerable interest but, to date, has produced limited clinical success. Multiple factors intrinsic to GBM biology help explain these disappointing outcomes. First, the GBM tumor microenvironment (TME) is potently immunosuppressive: tumor and stromal cells secrete TGF-β and other inhibitory mediators, recruit immunosuppressive myeloid populations, and upregulate checkpoint ligands, collectively creating barriers to effective antitumor T-cell responses [27,28]. Second, profound intratumoral heterogeneity and plasticity—including the presence of glioblastoma stem cells (GSCs) occupying protective niches such as perivascular and hypoxic zones—generate diverse cellular states that can evade immune recognition and repopulate the tumor after selective pressure [26,29,30,80]. Third, specific genetic alterations commonly found in GBM can shape immune resistance: for example, loss of PTEN has been linked to a more immunosuppressive microenvironment and reduced sensitivity to immune-based therapies. These features reduce neoantigen burden, impair antigen presentation, and limit productive T-cell infiltration, all of which blunt the efficacy of immune checkpoint inhibitors [20,27].

Mechanisms of resistance to checkpoint blockade in GBM are multifactorial and often overlapping. Antigenic heterogeneity and antigen loss lead to immune escape after targeted immune activation; immunosuppressive myeloid cells (TAMs) and regulatory populations can actively suppress effector T cells; impaired trafficking across the blood–brain barrier (and intratumoral stromal barriers) limits immune cell access; and adaptive resistance mechanisms upregulate alternative inhibitory pathways beyond PD-1/PD-L1, necessitating combinatorial blockade [26,27,28]. In addition, the low-to-moderate tumor mutational burden typical of many GBMs limits the abundance of neoantigens recognizable by the adaptive immune system, reducing the probability of durable responses to monotherapy checkpoint inhibitors [27,28].

Given these obstacles, contemporary efforts have shifted toward rational combination approaches designed to remodel the TME, increase antigen exposure, and produce more durable antitumor immunity. Oncolytic virotherapy represents a particularly attractive partner: oncolytic viruses can selectively lyse tumor cells, release tumor antigens in an inflammatory context, and reprogram the local immune milieu—thereby converting immunologically “cold” regions into “hot” ones and enhancing responsiveness to checkpoint blockade or other immunomodulators [82,85]. Early translational and preclinical work suggests that combining oncolytic platforms with immune stimulatory agents (e.g., cytokines, TLR agonists) or immune checkpoint inhibitors can potentiate antitumor T-cell activity and overcome some mechanisms of resistance, although these approaches still face delivery, safety, and regulatory challenges [82,85,87].

Other combinatorial strategies under active investigation include vaccines (personalized peptide or neoantigen vaccines) used to broaden the T-cell repertoire, and adoptive cellular therapies (e.g., CAR T cells) engineered to recognize GBM-associated antigens. Vaccination approaches have shown promise in inducing immune responses in selected patients, but clinical benefits are often transient, likely due to antigen heterogeneity and immune suppression within the TME [81]. CAR T-cell therapy in GBM has demonstrated feasibility in preclinical and early clinical studies, yet efficacy has been limited by antigen heterogeneity, on-target off-tumor toxicity concerns, and the hostile intratumoral environment; combination strategies that include cytokine support, epigenetic modulators, or local oncolytic vectors may be required to improve persistence and function of transferred cells [82,83].

To maximize the potential of immunotherapy in GBM, future trials should emphasize rational patient selection and biomarker-driven designs: identifying tumors with more permissive immune microenvironments, specific genetic alterations that predict benefit (or resistance), or particular methylation/epigenetic signatures could prioritize patients most likely to respond. Integration of single-cell and spatial profiling will be essential to understand the cellular and molecular determinants of response and resistance and to guide combinatorial regimens that target tumor cells, GSC niches, and immunosuppressive myeloid compartments simultaneously [26,79,80]. In summary, while monotherapy immune checkpoint inhibition has largely failed to deliver transformative benefit in unselected GBM cohorts, carefully designed combination strategies—particularly those that convert the TME, broaden antigen presentation, and counteract myeloid-mediated suppression—remain the most promising path forward [27,28,81,82,83,85,87].

Future therapeutic strategies for glioblastoma (GBM) are increasingly moving toward highly personalized, molecularly guided approaches supported by real-time adaptive clinical trial designs. Advances in genomic, epigenomic, and transcriptomic characterization—including single-cell and spatial profiling—have revealed the profound heterogeneity and dynamic plasticity of GBM, demonstrating that each tumor comprises multiple coexisting cellular states that evolve under therapeutic pressure [26,79,80]. This complexity underscores the need for individualized treatment concepts that incorporate a patient’s specific mutational profile, transcriptional program, epigenetic landscape, and immune microenvironment. Emerging personalized strategies include molecular subtype–based classification consistent with contemporary WHO CNS5 recommendations, which use integrated genomic and methylation signatures to guide prognosis and therapeutic choice [2,38]. Additionally, neoantigen-targeted vaccines and engineered cellular therapies, such as CAR T cells, are increasingly being designed to match patient-specific antigenic repertoires, aiming to overcome the challenges posed by antigenic heterogeneity and immunosuppression [81,82,83]. Insights into chromatin state, methylation patterns, and epigenetically driven signaling may further support the selection of synergistic combination therapies capable of reshaping tumor behavior and immune responsiveness [26,79].

As these individualized approaches become more sophisticated, progress in GBM therapy will increasingly depend on the implementation of adaptive clinical trial frameworks that allow investigators to modify therapeutic arms based on continuously updated biological and clinical data. Because GBM evolves rapidly and recurrent tumors often differ markedly from their initial presentation, adaptive designs provide a mechanism to align treatment decisions with the tumor’s current molecular profile rather than relying on static baseline assessments [25,80]. Such trial models can integrate serial multiomic monitoring, including repeat biopsies, liquid biopsy assays, and longitudinal single-cell or methylation analyses, enabling dynamic adjustment of treatment in response to emerging resistance mechanisms. This flexibility facilitates rational testing of combination therapies, such as pairing oncolytic viruses with checkpoint inhibitors or immune-stimulatory agents to remodel the tumor microenvironment, or selecting patients for vaccine-based or cellular immunotherapies on the basis of real-time antigen-expression patterns [81,82,83,85,87]. By uniting precision oncology with continuously adaptive therapeutic evaluation, these future directions aim to overcome the historical limitations of fixed-design trials and to better address the biological variability and evolutionary dynamics that define GBM. As multiomic technologies become increasingly accessible in clinical practice, such adaptive, personalized frameworks may ultimately offer a path toward more durable and meaningful treatment responses in a disease that has long resisted conventional therapeutic strategies. A summary of described therapies have been presented in Table 2.

## 6. Summary

Glioblastoma multiforme (GBM) is one of the most aggressive tumors of the central nervous system, characterized by high genetic heterogeneity, invasiveness, and resistance to treatment. Despite advances in molecular diagnostics, the prognosis remains poor, with a median survival of approximately 15 months. The introduction of the integrated WHO 2021 classification, combining histological and genetic assessment (including IDH, MGMT, TERT, EGFR, CDKN2A/B status) has enabled more precise diagnosis and prognosis. The development of modern methods such as NGS, radiogenomics, and liquid biopsy contributes to more accurate disease monitoring and represents a step towards precision medicine. The standard of care is still based on maximal resection, radiotherapy, and hepatemozolomide (Stupp regimen). However, frequent recurrences result from the presence of glioblastoma stem cells and the limited efficacy of drugs that cross the blood–brain barrier. In response, new therapies are being developed: immunotherapy (CAR-T, dendritic vaccines), virotherapy, Tumour Treating Fields, and drug nanocarriers. The future of GBM treatment involves further personalization of therapy, integration of molecular, imaging, and clinical data, and the use of artificial intelligence. Such an interdisciplinary, adaptive approach offers an actual chance to improve the prognosis and quality of life of patients with this exceptionally malignant tumor.

## Figures and Tables

**Table 1:** The most common clinical features and course of glioblastoma (GBM).

Symptom/Feature	Clinical Description	References
Paresis, aphasia	Focal neurological deficits depending on tumor location	[32,33]
Seizures	Common, especially with cortical involvement	[32]
Headache, nausea	Symptoms of increased intracranial pressure	[32]
Cognitive changes	Memory and personality disturbances, disorientation	[32]
Rapid progression, recurrence	High mortality and treatment resistance	[32,35,36]

**Table 2:** Summary of therapeutic strategies in glioblastoma multiforme.

Therapy/Agent	Mechanism	Trial Phase	Key Limitations
Standard radiotherapy + temozolomide	DNA damage, cytotoxic effect	Standard of care	Limited efficacy in MGMT unmethylated patients; tumor recurrence common
Tumor Treating Fields (TTFields, Optune)	Disrupt mitosis via alternating electric fields	Approved	Availability, compliance, cost; long-term data limited
Personalized neoantigen vaccines	Induce patient-specific T-cell responses	Early-phase	Survival benefit unconfirmed; glucocorticoid effect; antigen heterogeneity
CAR-T cells (IL13Rα2, EGFRvIII, EGFR)	Target GBM-associated antigens via adoptive transfer	Early clinical	Transient responses; antigen heterogeneity; immunosuppressive TME
BiTE molecules (EGFRvIII/EGFR)	Engage T cells with tumor cells	Preclinical/Early-phase	Limited clinical data; effect durability unknown
Oncolytic viruses (DNX-2401, PVSRIPO, Delta-24-RGD)	Tumor lysis + immune activation	Phase I/II	Delivery and safety challenges; transient efficacy
Nanoparticle/liposome-based delivery	Targeted cytostatic/oligonucleotide delivery	Preclinical/Early-phase	Distribution, elimination, immunogenicity issues
Stem cell-based therapies	Tumor-tropic delivery of prodrugs/gene vectors	Preclinical/Early-phase	Safety (transformation potential); function control
Heparanase inhibitor (RDS 3337)	Shift autophagy-apoptosis balance toward apoptosis	Preclinical	Clinical translation unproven; pharmacokinetics and safety require study

## Data Availability

No new data were created or analyzed in this study. Data sharing is not applicable to this article.
